# The stromal tumor-infiltrating lymphocytes, cancer stemness, epithelial-mesenchymal transition, and B7-H4 expression in ovarian serous carcinoma

**DOI:** 10.1186/s13048-022-01076-z

**Published:** 2023-01-06

**Authors:** Chungsu Hwang, Hyun Jung Lee, Ju-Young Na, Ki Hyung Kim, Yong Jung Song, Jee Yeon Kim, Kyungbin Kim, Dong Hoon Shin, Joon Young Park, So Young Kim, Jung Hee Lee, Kyung Un Choi

**Affiliations:** 1 Research Institute for Convergence of Biomedical Science and Technology, Pusan National Yangsan Hospital, 20, Geumo-Ro, Mulguem-Eup, Yangsan-Si, Gyeongsangnam-Do South Korea; 2 Department of Pathology, Pusan National University Yangsan Hospital, 20, Geumo-Ro, Mulguem-Eup, Yangsan-Si, Gyeongsangnam-Do South Korea; 3 Department of Obstetrics and Gynecology, Pusan National University Hospital, 179 Gudeok-Ro, Seo-Gu, Busan, 49241 South Korea; 4 Department of Obstetrics and Gynecology, Pusan National University Yangsan Hospital, 20, Geumo-Ro, Mulguem-Eup, Yangsan-Si, Gyeongsangnam-Do South Korea; 5 Department of Pathology, Pusan National University Hospital and Pusan National University School of Medicine, 179 Gudeok-Ro, Seo-Gu, Busan, 49241 South Korea

**Keywords:** B7-H4, Ovarian serous carcinoma, Tumor-infiltrating lymphocytes, Cancer stem cell, Epithelial-mesenchymal transition

## Abstract

**Background:**

B7-H4 is expressed in various types of cancers and its expression inversely correlates with the degree of tumor-infiltrating lymphocytes (TILs). Studies have shown the relationship between B7-H4, cancer stem cell (CSC) properties, and epithelial-mesenchymal transition (EMT) in various cancers. However, very few studies have investigated the relationship between B7-H4, TILs, cancer stemness, and EMT in epithelial ovarian cancer (EOC). The present study aimed to elucidate whether B7-H4 is involved in immune evasion and examine whether B7-H4 is associated with cancer stemness or EMT in ovarian serous carcinoma, the most common type of EOC. The clinical significance of B7-H4 was also investigated to evaluate its potential as a therapeutic target.

**Methods:**

A total of 145 patients included in this study. The degree of stromal TILs was evaluated using hematoxylin and eosin (H&E)-stained slides. Immunohistochemical analysis of B7-H4, CSC-related biomarkers (CD24, CD44s, CD133, and ALDH1), and EMT-related biomarkers (E-cadherin, N-cadherin, and vimentin) was performed using tissue microarray. qRT-PCR for *VTCN1*, *CD24*, *CD44*, *PROM1*, *ALDH1*, *CDH1*, *CDH2*, and *VIM* genes was performed on 38 frozen tissue samples. The mRNA expression levels were analyzed using Gene Expression Profiling Interactive Analysis (GEPIA) online analysis tool.

**Results:**

B7-H4 protein expression positively correlated with the degree of stromal TILs. CD24, CD44s, and CD133 expression showed a positive correlation with B7-H4 expression at both the protein and mRNA levels, but ALDH1 correlated only at the protein level. E-cadherin expression was positively correlated with B7-H4 expression at both the protein and mRNA levels. N-cadherin and vimentin expression was inversely related to B7-H4 expression only at the mRNA level. B7-H4 positive patients were associated with higher tumor grade and lower overall survival rate than B7-H4 negative patients, especially in ovarian serous carcinoma with low stromal TILs.

**Conclusions:**

The present study demonstrates that B7-H4 may not be involved in the immune evasion mechanism, but is involved in cancer stemness and mesenchymal-epithelial transition. In addition, B7-H4 may be a therapeutic target for the treatment of ovarian serous carcinoma, especially with low stromal TILs.

**Supplementary Information:**

The online version contains supplementary material available at 10.1186/s13048-022-01076-z.

## Introduction

Epithelial ovarian cancer accounts for 2.5% of all female malignancies, but is the cause for 5% of cancer-related deaths in women [1]. Although cytoreductive surgery followed by chemotherapy is the standard treatment for ovarian epithelial cancer, the mortality rates have not improved markedly. Therefore, new therapies and prognostic methods need to be developed for patients with EOC.

PD-L1 is expressed in various cancers, and attenuates the host immune reaction to cancer cells. Several studies have shown that blocking the interaction between PD-1 and PD-L1 increases T cell activity against tumor cells [2, 3]. Clinical trials have shown that various types of human malignancies regress completely or partially following treatment with PD-1 or PD-L1 inhibitors [4–6]. The US Food and Drug Administration has approved a few PD-1/PD-L1 inhibitors, such as duvalumab and pembrolizumab, for the treatment of patients with non-small cell carcinoma or urothelial carcinoma.

However, accumulating data show that the response rates to anti-PD-1 or anti-PD-L1 antibodies are relatively low, and it is uncertain whether PD-L1 expression predicts the degree of response to PD-1/PD-L1 inhibitors in EOC [7–10]. Our previous study showed that PD-L1 expression on tumor cells was not associated with the overall survival rate of patients with EOCs, although stromal PD-L1 expression was an independent prognostic factor, especially in ovarian serous carcinoma [11]. These data suggest that PD-L1 expression in ovarian cancer cells may not be involved in the host immune evasion mechanism, and it is necessary to study whether other members of the B7 family proteins are related to the host immune evasion mechanism in EOC.

B7-H4 is a member of the B7 family proteins that was discovered recently. B7-H4 protein is expressed in T cells, B cells, monocytes, and dendritic cells following in vitro stimulation. It acts as a co-inhibitory factor that suppresses cytokine production and cell cycle processes in T cells and thus inhibits T cell immune response [12, 13]. Recent studies have shown that a variety of cancers express B7-H4 [14–16]. The association between B7-H4 expression and the degree of TILs has been examined in various cancers, and B7-H4 expression levels were reported to be inversely associated with the degree of TILs in most studies [17–20]. These results suggest that cancers may evade the host immune system by expressing the B7-H4 protein. B7-H4 also can be expressed on EOC [21]. Some studies have identified the relationship between B7-H4 expression in EOC and the degree of TILs [22, 23]. However, there is insufficient data to deduce the immunological function of B7-H4 in EOC, and the results have been inconsistent. In addition, B7-H4 may be involved in CSC or epithelial-to-mesenchymal transition (EMT)-related pathways based on the results of studies in various cancers, including esophageal squamous cell carcinoma, colorectal cancer, intrahepatic cholangiocarcinoma, and bladder cancer [24–27]. However, there is no research on the association between B7-H4 and CSC or EMT in EOC.

This study aimed to compare B7-H4 expression level and the density of stromal TILs in ovarian serous carcinoma, the most common EOC to determine whether B7-H4 is associated with immune evasion mechanism. To identify whether B7-H4 is related to CSC or EMT-related pathways, B7-H4 expression levels were compared with that of CSC and EMT-related biomarkers. The relationship between B7-H4 expression and clinicopathologic factors, and the effect of B7-H4 on survival rate also were also investigated to determine the clinical significance and the potential of B7-H4 as a therapeutic target for ovarian serous carcinoma.

## Materials and methods

### Patient selection

The study was conducted on 145 patients who underwent exploratory laparotomy for ovarian serous carcinoma in the Department of Obstetrics and Gynecology of the Pusan National University Hospital (PNUH) between 1998 and 2013. All patients provided written informed consent prior to undergoing the surgical procedures. The biospecimens used in this study were provided by the Ministry of Health and Welfare. All samples derived from the National Biobank of Korea were obtained with approval from the institutional review board.

Tumors were histologically diagnosed according to the World Health Organization (WHO) classification, and tumor stage was determined according to the International Federation of Gynecology and Obstetrics (FIGO) criteria. Tumor grade, mitosis, and nuclear grade were evaluated using a microscope. Other clinical data, such as residual tumor, tumor stage, and response to chemotherapy were collected from the electronic medical records of PNUH. Nuclear grade was reclassified into two groups by grouping mild and moderate into one group. Residual tumor was classified as optimal if the size of the remaining tumor was ≤ 1.0 cm, and suboptimal if it was ≥ 1.0 cm. Tumor stage was reclassified as early stage for stage I and advanced stage for stages II, III, and IV. Chemoresponse was reclassified as regressive disease for complete or partial regression and stable/progressive disease for stable disease or progression. Overall survival was measured from diagnosis to death or until the last follow-up visit. All patients were followed-up successfully.

### Evaluation of stromal TILs

Stromal TIL values were obtained from our previous study [28]. Stromal TILs were evaluated according to the recommendations of the International TILs Working Group 2014 [29]. The degree of stromal TILs was reported as a percentage of the inflammatory cells in the stromal area, and the percentage of stromal TILs was evaluated via eye measurement. Although the International TILs Working Group 2014 recommends that representative slides are sufficient to determine the stromal TIL value in each case, all available slides for each case were evaluated. The average value was taken as the stromal TIL value. Stromal TILs in the tumor areas with crush artifacts, necrosis, or hyalinization were excluded. Polymorphonuclear leukocytes were also excluded from the study. The representative cases of low and high stromal TILs are shown in supplementary figure S1.

### Tissue microarray construction and immunohistochemistry

Two representative portions of the tumor were selected and annotated on H&E-stained slides. Two cores (2 mm in diameter) were collected from the same area of formalin-fixed and paraffin-embedded blocks and re-embedded in the recipient paraffin blocks to construct tissue microarray blocks. CD24, CD44s, CD133, and ALDH1 were chosen as CSC-related biomarkers. E-cadherin, N-cadherin, and vimentin were chosen as EMT-related biomarkers. Tissue microarray blocks were sectioned at 4 µm thickness and immunostained for B7-H4, CD24, CD44s, CD133, ALDH1, E-cadherin, N-cadherin, and vimentin using a Bond-Max Autostainer (Leica Microsystems, Bannockburn, IL, USA) according to the manufacturer’s protocol. Information on the antibodies used is provided in Table 1.

**Table 1:** Details of the antibodies used in this study

Biomarkers	Clone	Dilution	Company
B7-H4	D1M81	1:100	Cell Signaling Technology
CSC-related biomarkers			
CD24	SN3	1:100	Invitrogen
CD44s	156-3C11	1:100	Cell Signaling Technology
CD133	Ab19898	1:100	Abcam
ALDH1	44/ALDH	1:100	BD biosciences
EMT-related biomarker			
E-cadherin	4A2C7	1:200	Invitrogen
N-cadherin	3B9	1:100	Invitrogen
Vimentin	V9	1:100	Invitrogen

### Evaluation of immunostaining

**Fig. 1:**
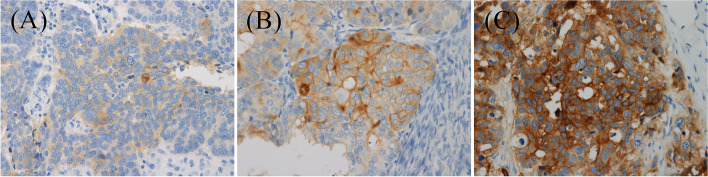
Representative cases of B7-H4 immunohistochemical expression in tumor cell membrane and cytoplasm with mild intensity (**A**, magnification, × 400), moderate intensity (**B**, magnification, × 400), and marked intensity (**C**, magnification, × 400)

The tumor cells that were immunostained for B7-H4 on the membrane or cytoplasm were considered positive. The intensity and proportion of B7-H4 on tumor cells were evaluated, and the Allred scoring system was used to grade the degree of B7-H4 immunostaining [30]. The intensity of B7-H4 staining on tumor cells in each core was graded on a semi-quantitative scale of 0 (none), 1 + (mild), 2 + (moderate), and 3 + (marked). A representative case of each intensity is shown in Fig. 1. The final intensity for each patient was determined as the intensity with the highest proportion among the two cores. The proportion of B7-H4 on tumor cells in each core was defined as the ratio of the area of the tumor cells expressing B7-H4 to the entire tumor area in the TMA core, and the final proportion for each patient was defined as the average proportion of the two cores. The final proportion for each patient was semi-quantitatively categorized as: 0 (0% proportion), 1 (< 1% proportion), 2 (1–10% proportion), 3 (11–33% proportion), 4 (34–66% proportion), and 5 (67–100% proportion). The sum of the intensity and the proportion score formed the final immunostaining score, and a score of 0–1 was considered negative and score of 2–8 was considered positive.

All CSC-related biomarkers were immunostained in the cytoplasm or membrane of the cancer cells (Supplementary figure S2). Among the EMT-related biomarkers, E-cadherin and vimentin showed membranous immunostaining patterns. N-cadherin was immunostained in the cytoplasm of cancer cells (Supplementary figure S3). The intensity of all biomarkers in tumor cells in each core was graded on a semi-quantitative scale of 0 (non), 1 + (mild), 2 + (moderate), and 3 + (marked), and the final intensity was determined in a manner identical to that used for B7-H4 evaluation. The proportion of each biomarker was evaluated, and the final proportion was determined in a manner identical to that used for the B7-H4 evaluation.

### RNA extraction and quantitative real-time polymerase chain reaction (qRT-PCR)

qRT-PCR was performed on 38 frozen tissue specimens of ovarian serous carcinoma. Total RNA was extracted from the frozen tissues using the TRIzol reagent (Invitrogen, USA). Reverse transcription was performed with total RNA using SuperScript™ II Reverse Transcriptase (Invitrogen), according to the manufacturer’s instructions. The cDNA derived from the mRNA was amplified using primer pairs. Real–time PCR was performed on the StepOnePlus™ Real–Time PCR System (Applied Biosystems, USA) using SYBR® Green PCR Kit (Applied Biosystems, USA), according to the manufacturer’s instructions. The thermal cycling conditions were 95 ℃ for 15 s and 30 s at the optimal temperature (59 ℃). The data were analyzed using StepOne software v2.2.2 (Applied Biosystems, USA). The expression level of each mRNA was normalized to that of an endogenous control reference gene (GAPDH) and calculated using the 2^−△△Ct^ method. Primer sequences for GAPDH and each gene are listed in Table 2.

**Table 2:** Forward and reverse primers used for qRT-PCR

Gene	Forward primer	Reverse primer
VTCN1	AAAAGGCGGAGTCACCTACA	CCAGCTGATGGCAAAGAAAG
CD24	CTGCAGTCAACAGCCAGTCT	ACGTTTCTTGGCCTGAGTCT
CD44	CTCTTGGCCTTGGCTTTGAT	TCTTCTGCCCACACCTTCTT
PROM1	CAGCAACGAGTCCTTCCTAT	CCCTGTGCGTTGAAGTATCT
ALDH1A	GGCTTATCAGCAGGAGTGTT	GCAATTCACCCACACTGTTC
VIM	GAGATTGCCACCTACAGGAA	TTCAGGGAGGAAAAGTTTGG
CDH1	ACACCCGGGACAACGTTTAT	GTGCAGCTGGCTCAAGTCAA
CDH2	GGGTCCTTGAGCTCCCTTAA	CCCAGTCGTTCAGGTAATCA
GAPDH	CGAGATCCCTCCAAAATCAA	CCTTCTCCATGGTGGTGAA

### Gene expression profiling interactive analysis (GEPIA) online analysis tool

GEPIA is a web-based tool that provides customizable functions including differential expression analysis, profiling plotting, correlation analysis, patient survival analysis, similar gene detection, and dimensionality reduction analysis based on The Cancer Genome Atlas (TCGA) and Genotype-Tissue Expression (GTEx) data (http://gepia.cancer-pku.cn/). We used the correlation analysis function of the GEPIA online analysis tool to analyze mRNA expression data of *VTCN1, CD24, CD44, PROM1, ALDH1A1, CDH1, CDH2*, and *VIM* genes.

### Statistical analysis

Spearman’s correlation test was used to identify the relationship between the mRNA and immunohistochemical expression levels of B7-H4 and the density of stromal TILs. The correlation between mRNA and immunohistochemical expression of B7-H4 and those of CD24, CD44s, CD133, ALDH1, E-cadherin, N-cadherin, and vimentin were identified using Spearman’s correlation test. Chi-square and Fisher’s exact tests were used to identify the relationship between the patient groups based on B7-H4 expression level and clinicopathologic factors. The effect of B7-H4 expression on the overall survival rate was evaluated using the Kaplan–Meier curve analysis and log-rank test. Statistical analyses were performed using the R version 4.0.2. Statistical significance was set at *P*-value < 0.05.

## Results

### Characteristics of the patients

The mean age of the patients was 57.9 years (range, 33–79 years) and the follow-up period ranged from 1 to 208 months (median follow-up period, 62 months). The patients included 12 (8.3%) with grade 1, 81 (55.9%) with grade 2, and 52 (35.8%) with grade 3 ovarian serous carcinoma. Among these, 72 (49.7%) of 145 cases were diagnosed with mild and moderate nuclear atypia, and 73 (49.7%) cases showed marked nuclear atypia. Of the 145 patients, 32 (22.1%) had 0–9 mitosis/10 HPFs, 61 (42.1%) had 10–24 mitosis/10 HPFs, and 52 (35.8%) had > 24 mitosis/10 HPFs. Due to lack of medical records, residual tumor and tumor stage was confirmed only in 105 out of 145 patients. Of the 105 patients, 93 (88.6%) underwent optimal debulking surgery, and 12 (11.4%) underwent suboptimal debulking surgery. In addition, 21 (20.0%) patients were diagnosed with early-stage disease, and 84 (80.0%) patients with advanced-stage disease. Excluding patients who did not receive chemotherapy or whose chemotherapy records could not be identified, 45 (46.4%) of 97 patients showed complete or partial regression, while 52 (53.6%) had stable or progressive disease (Table 3).

**Table 3:** The clinicopathologic characteristics of the patients

Clinicopathologic factors	Number of patients (%)
Tumor grade	
1	12 (8.3)
2	81 (55.9)
3	52 (35.8)
Nuclear grade	
Mild and moderate	72 (49.7)
Marked	73 (50.3)
Mitosis	
0–9/10 HPFs	32 (22.1)
10–24/10 HPFs	61 (42.1)
≥ 25/10 HPFs	52 (35.8)
Residual tumor	
Optimal	93 (88.6)
Suboptimal	12 (11.4)
Tumor stage	
Early	21 (20.0)
Advanced	84 (80.0)
Chemoresponse	
Regressive disease	45 (46.4)
Stable/progressive disease	52 (53.6)
Stromal TILs	
Low stromal TIL group	112 (77.2)
High stromal TIL group	33 (22.8)

### Association between B7-H4 and stromal TILs

**Fig. 2:**
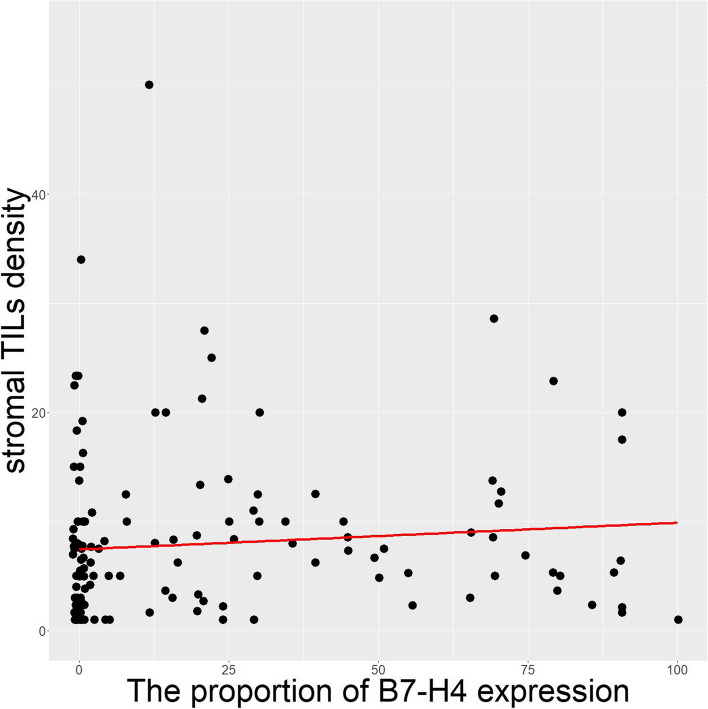
Scatter plot for the relationship between the proportion of B7-H4 immunohistochemical expression and the density of stromal TILs (*p* = 0.008, rho = 0.226)

The average stromal TIL value of all 145 patients was 8.06%. The average number of slides evaluated was 4.6. Patients were classified into the low stromal TIL group if the stromal TIL value was the ≤ 10%, or high stromal TIL group if the stromal TIL value was > 10%. In total, 112 (77.2%) out of 145 patients were classified into low- and 33 (22.8%) into high-stromal TIL groups (Table 3). To identify the relationship between B7-H4 protein expression and the density of stromal TILs, we performed Spearman’s correlation test using the proportion of B7-H4 immunostaining and the density of stromal TILs. The proportion of positive B7-H4 immunostaining showed a significant positive correlation with the density of stromal TILs (*p* = 0.008, rho = 0.226) (Fig. 2).

### Association between B7-H4 and CSC-related biomarkers

**Fig. 3:**
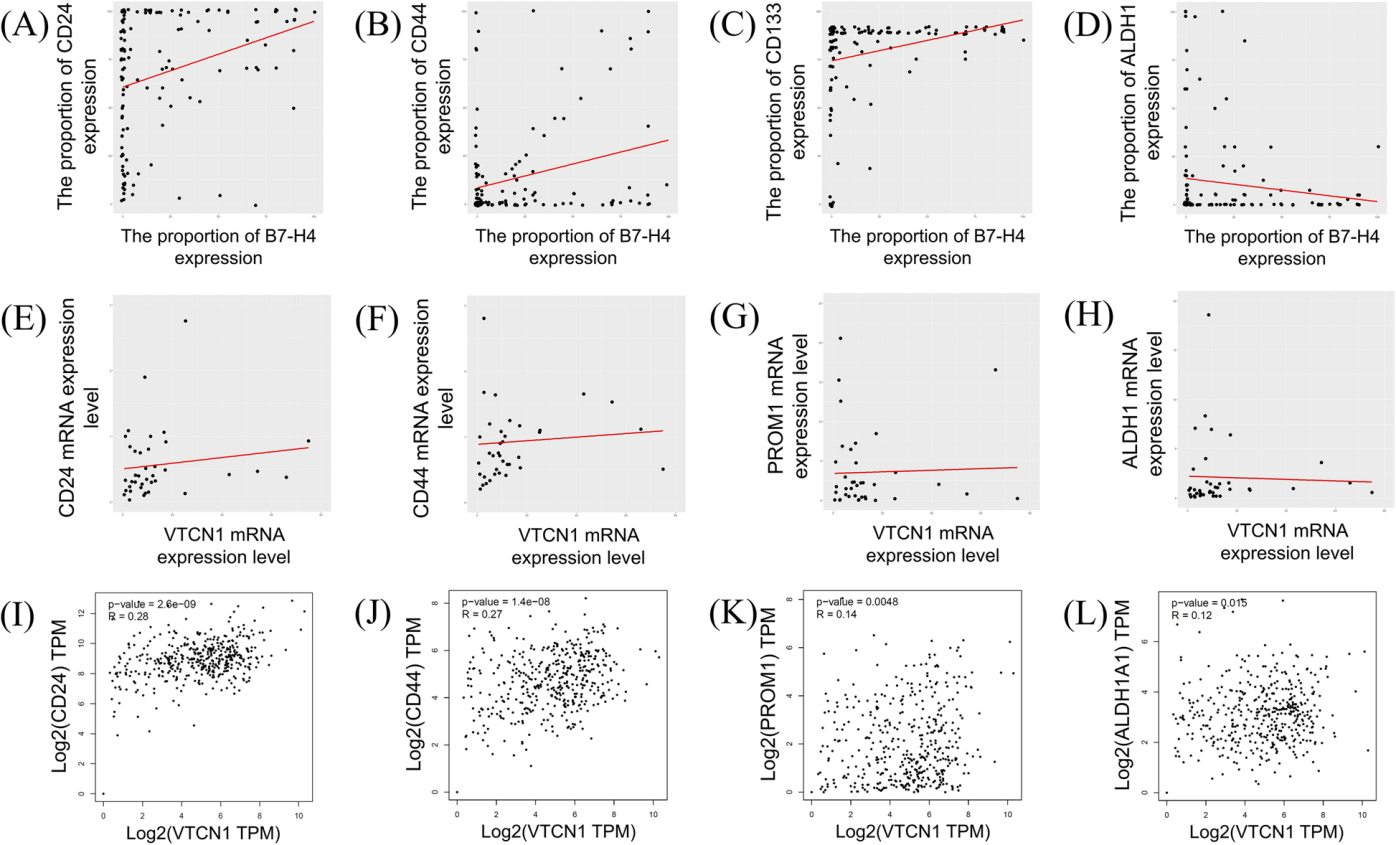
Scatter plots for the relationship between the expression of B7-H4 and CSC-related biomarkers. **A-D** Scatter plots using the proportion of immunohistochemical expression. **A**, CD24; **B**, CD44s; **C**, CD133; **D**, ALDH1. (**E–H**) Scatter plots using mRNA expression data from the frozen tissues. **E**, *CD24*; **F**, *CD44*; **G**, *PROM1*; **H**, *ALDH1*. **I-L** Scatter plots using mRNA expression data from the GEPIA online analysis tools **I**, *CD24*; **J**, *CD44*; **K**, *PROM1*; **L**, *ALDH1*

Spearman’s correlation test was performed using the proportions of B7-H4, CD24, CD44s, CD133, and ALDH1 immunohistochemical expression. The results showed that the expression of B7-H4 was positively associated with expression of CD24 (*p* < 0.001, rho = 0.313), CD44s (*p* = 0.004, rho = 0.249), and CD133 (*p* < 0.001, rho = 0.286) (Fig. 3A-C, respectively). No significant correlation was identified between B7-H4 and ALDH1 immunohistochemical expression (*p* = 0.479, rho = -0.061) (Fig. 3D). Spearman’s correlation test, performed using mRNA expression data from the 38 frozen tissue specimens, showed that *VTCN1* mRNA expression level did not significantly correlate with that of any CSC-related gene (Fig. 3E–H). However, using mRNA expression data from the GEPIA online analysis tool, the mRNA expression levels of all CSC-related genes showed a significant positive correlation with that of *VTCN1* (Fig. 3I–L). The results of Spearman's correlation test are listed in Table 4.

**Table 4:** The results of Spearman's correlation test between B7-H4 and all biomarkers

Biomarkers	B7-H4 Immunohistochemistry		*VTCN1*				
			Biomarkers	qRT-PCR		GEPIA dataset	
	rho	*p*-value		rho	*p*-value	rho	*p*-value
CD24	0.313	** < 0.001**	*CD24*	0.271	0.100	0.28	** < 0.001**
CD44s	0.249	**0.004**	*CD44*	0.256	0.121	0.27	** < 0.001**
CD133	0.286	** < 0.001**	*PROM1*	-0.098	0.558	0.14	**0.005**
ALDH1	-0.061	0.479	*ALDH1*	0.23	0.164	0.12	**0.015**
E-cadherin	0.261	**0.002**	*CDH1*	0.357	**0.029**	0.18	** < 0.001**
N-cadherin	-0.021	0.818	*CDH2*	-0.329	**0.044**	-0.34	** < 0.001**
vimentin	-0.141	0.1	*VIM*	0.057	0.733	-0.11	**0.029**

### Association between B7-H4 and EMT-related biomarkers

**Fig. 4:**
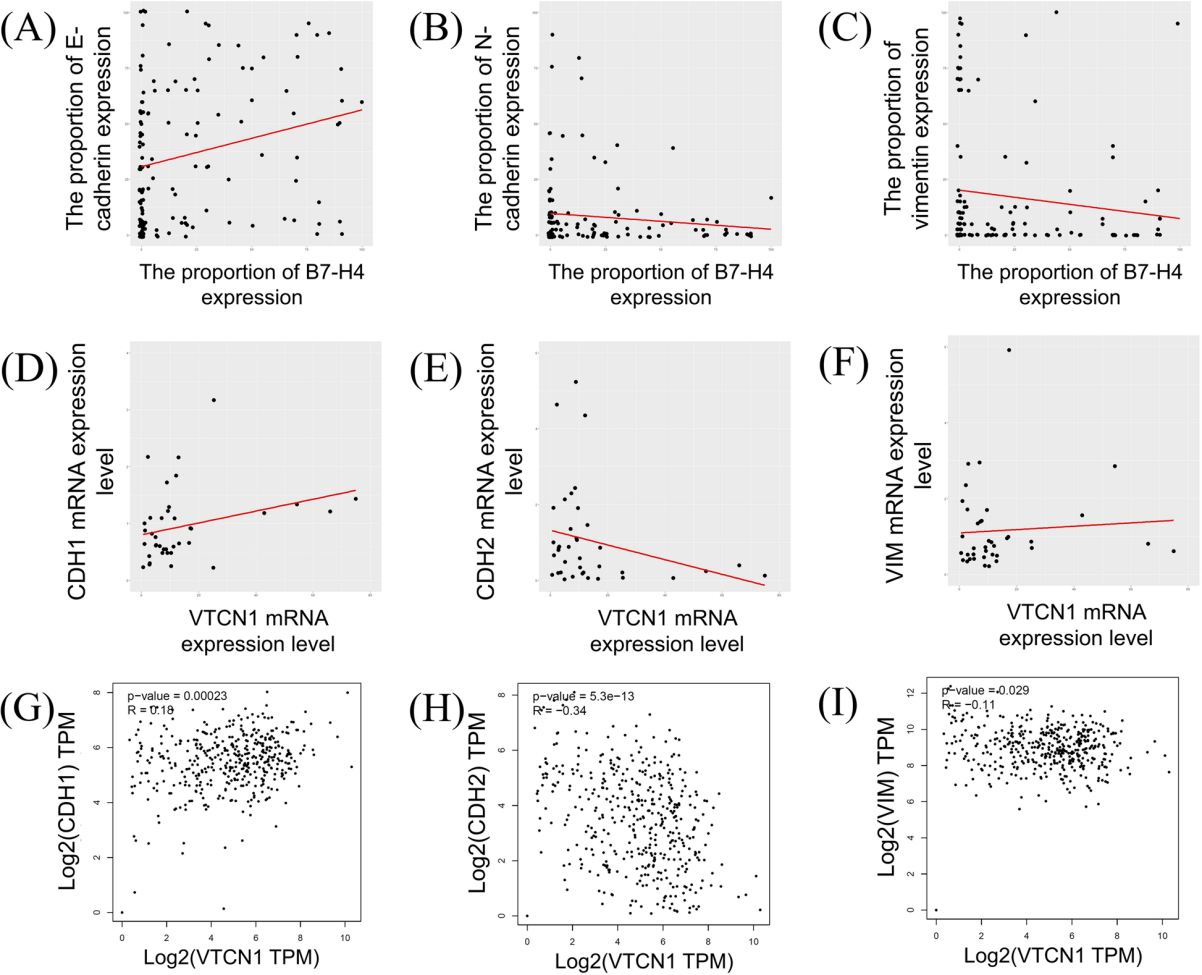
Scatter plots for the relationship between the expression of B7-H4 and EMT-related biomarkers. **A-C** Scatter plots using the proportion of immunohistochemical expression. **A**, E-cadherin; **B**, N-cadherin; **C**, vimentin. **D-F** Scatter plots using mRNA expression data from the frozen tissues. **D**, *CDH1*; **E**, *CDH2*; **F**, *VIM*. **G-I** Scatter plots using mRNA expression data from the GEPIA online analysis tools **G**, *CDH1*; **H**, *CDH2*; **I**, *VIM*

Spearman’s correlation test revealed that the proportion of B7-H4, detected immunohistochemically, was significantly positively correlated with that of E-cadherin (*p* = 0.002, rho = 0.261) (Fig. 4A). However, the proportion of N-cadherin and vimentin was not significantly correlated with that of B7-H4 (*p* = 0.818, rho = -0.021 and *p* = 0.100, rho = -0.141, respectively) (Fig. 4B and C). Analysis of expression data from the 38 frozen tissue specimens revealed that *VTCN1* mRNA expression level was positively correlated with that of *CDH1* (*p* = 0.029, rho = 0.357) and inversely correlated with that of *CDH2* (*p* = 0.044, rho = -0.329) (Fig. 4D and E). Analysis of mRNA expression data from the GEPIA online analysis tool revealed that *CDH1* mRNA expression level was positively correlated with that of *VTCN1* (*p* < 0.001, rho = 0.180) (Fig. 4G). Both *CDH2* and *VIM* mRNA expression levels were inversely correlated with that of *VTCN1* (*p* < 0.001, rho = -0.340 and *p* = 0.029, rho = -0.110, respectively) (Fig. 4H and I). The results of Spearman's correlation test are listed in Table 4.

### Clinical significance of B7-H4

Among the 145 cases, B7-H4 expression was immunohistochemically confirmed in all (137 cases) but eight cases in which both the cores could not be evaluated due to damage incurred during the process of cutting the TMA blocks or due to uneven staining. Using the Allred scoring system, 75 (54.7%) out of 137 patients were included in the B7-H4 positive group and 60 (45.3%) in the B7-H4 negative group. To confirm the relationship between the patient groups and clinicopathological factors, Chi-square and Fisher’s exact tests were performed. Tumor grade was significantly associated with the B7-H4 positive group. Patients in the B7-H4 positive group had a higher tumor grade than those in the B7-H4 negative group (*p* = 0.017). However, there was no relationship between the patient groups and other clinicopathological factors (Table 5).

**Table 5:** Association between the B7-H4 expression groups and clinicopathologic factors in ovarian serous carcinoma

Clinicopathologic factors	B7-H4 negative group	B7-H4 positive group	*p*-value
Residual tumor			0.095
Optimal	47 (53.4)	41 (46.6)	
Suboptimal	2 (20.0)	8 (80.0)	
Tumor grade			**0.017**
1	10 (83.3)	2 (16.7)	
2	33 (44.0)	42 (56.0)	
3	19 (38.0)	31 (62.0)	
Tumor stage			1.000
Early	9 (47.4)	10 (52.6)	
Advanced	40 (50.6)	39 (49.4)	
Nuclear grade			0.151
Mild and moderate	35 (52.2)	32 (47.8)	
Marked	27 (38.6)	43 (61.4)	
Mitosis			0.558
0–9/10 HPFs	16 (51.6)	15 (48.4)	
10–24/10 HPFs	27 (46.6)	31 (53.4)	
≥ 25/10 HPFs	19 (39.6)	29 (60.4)	
Chemoresponse			0.418
Regressive disease	22 (53.7)	19 (46.3)	
Stable/progressive disease	21 (42.9)	28 (57.1)	

**Fig. 5:**
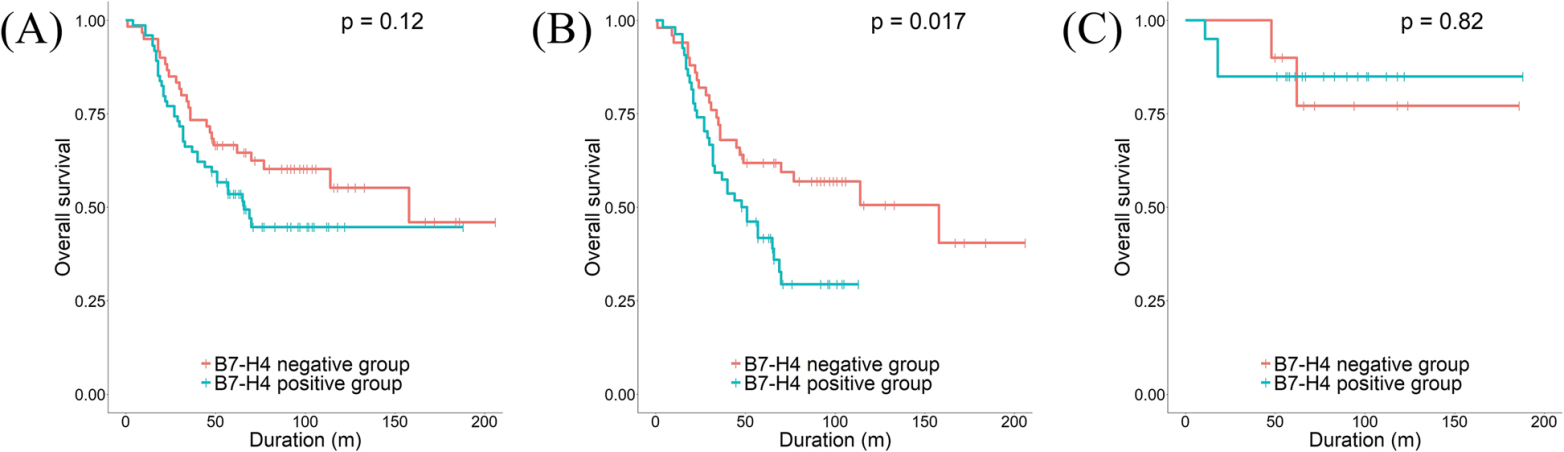
Results of Kaplan–Meier curve analysis and log rank test for overall survival between the patient groups in all ovarian serous carcinoma (**A**, *p* = 0.120), ovarian serous carcinoma with low stromal TILs (**B**, *p* = 0.017), and ovarian serous carcinoma with high stromal TILs (**C**, *p* = 0.820)

The effect of B7-H4 expression on overall survival rate was evaluated using Kaplan–Meier curve analysis and log-rank test. Although the B7-H4 positive group tended to have lower overall survival rate, the p-value was not significant in the log-rank test (*p* = 0.120) (Fig. 5A). However, the B7-H4 positive group was associated with a significant decrease in the overall survival rate of patients with low stromal TILs (*p* = 0.017) (Fig. 5B), but not of patients with high stromal TILs (*p* = 0.820) (Fig. 5C).

## Discussions

Our study showed that the proportion of B7-H4 immunohistochemical expression was positively correlated with the degree of stromal TILs in ovarian serous carcinoma.

Pagnotti et al. performed the only study that investigated the relationship between B7-H4 protein level and the degree of TILs in ovarian serous carcinoma. Unlike the results of our study, they showed that B7-H4 protein level was not associated with the degree of infiltration of CD3 + , CD4 + , CD8 + , and CD14 + lymphocytes in ovarian serous carcinoma [23]. However, there are some differences in the methodologies used in the study by Pagnotti et al. and ours. Whereas 145 cases of ovarian serous carcinoma were included in our study, only 12 cases were used in the study by Pagnotti et al. Therefore, the data from our study is statistically more reliable than that from the study by Pagnotti et al.. Furthermore, Pagnotti et al. selected the three most severe parts of the TILs to determine the degree of TILs, whereas we evaluated TILs in all H&E-stained slides for each case, and used the average value as the degree of TILs in each case. Our previous study showed that evaluating as many slides as possible improved the accuracy of TIL measurements compared to evaluating the severe parts only [28]. The type of inflammatory cells that are measured also influences the results. We measured the degree of general inflammatory cell infiltration by evaluating H&E-stained slides, whereas Pagnotti et al. evaluated each type of inflammatory cell using immunohistochemical staining.

The positive correlation between B7-H4 expression and the degree of stromal TILs indicated that B7-H4 may not be involved in the host immune evasion mechanism. MacGregor et al. did not find any difference between the number of T and B cells, and T cell phenotype between high B7-H4-expressing and low B7-H4-expressing tumor. In addition, there was no relationship between B7-H4 expression and that of inhibitory ligands such as PD-1, TIM3, and LAG3 [22]. Therefore, they concluded that B7-H4 is not associated with an inhibitory microenvironment, which is in agreement with our results. Our results also suggested that B7-H4 expression in tumor cells may be induced by the inflammatory microenvironment. The expression of some immune-related molecules, such as IL-4, IL-6, IL-10, and GM-CSF, were analyzed, but none of them was found to be related to the induction of B7-H4 expression in ovarian cancer cells [31]. Further studies are needed to elucidate the relationship between inflammatory microenvironment and the induction of B7-H4 expression in tumor cells.

Although many studies have reported that B7-H4 expression levels are inversely correlated with the density of TILs in various cancer types, a few cancers did not show this correlation. For example, in small cell lung cancer, B7-H4 expression did not show any correlation with the degree of infiltration of CD3 + , CD8 + , and CD20 + TILs, but increased B7-H4 expression was associated with a low 5-year overall survival rate [32]. In our study, the patient group with positive B7-H4 expression had a higher tumor grade and tended to have a lower overall survival rate than the patient group with negative B7-H4 expression. These results provide further evidence that B7-H4 may play different roles in the prognosis of patients depending on the type of cancer.

Some studies have shown that B7-H4 expression in tumor cells is related to CSC-properties. Kang et al. reported that the inhibition of B7-H4 expression in hepatocellular carcinoma cell lines resulted in a decrease in the CD44 + /CD133 + double-positive cell population [33]. According to a study by Piao et al. using esophageal squamous cell carcinoma, increased B7-H4 expression level was associated with increased expression of cancer stemness-related proteins, such as Sox9, LSD1, Oct4, and LGR4 [24]. However, studies investigating the relationship between B7-H4 and cancer stemness are limited, and no study has reported the relationship between B7-H4 and CSC-related biomarkers in EOC.

This is the first study to demonstrate the association between the expression levels of B7-H4 and CSC-related biomarkers in ovarian serous carcinoma. Immunohistochemical expression of B7-H4 showed a significant positive correlation with that of CD24, CD44s, and CD133. Increased *VTCN1* mRNA expression from the GEPIA online analysis tool was also associated with increased CSC-related gene expression. However, when in the mRNA expression data from frozen tissue specimens, *VTCN1* mRNA expression showed no association with any CSC-related genes. This may be because the number of frozen tissue specimens was too small to obtain a statistical significance. CD24, CD44s, CD133, and ALDH1 are biomarkers widely used to identify CSCs in EOCs [34]. The results indicated that B7-H4 may be correlated with the cancer stemness of ovarian serous carcinoma. The association between B7-H4 and cancer stemness may explain why high-grade tumors and low overall survival rates were observed in the B7-H4-positive ovarian serous carcinomas.

Latifi et al. demonstrated that treatment of ovarian cancer cell lines with cisplatin resulted in the upregulation of both EMT- and CSC-related biomarkers via Erk pathway [35]. Lupia et al. reported that both CSC- and EMT-related biomarkers are simultaneously regulated by CD73 in ovarian cancer [36]. These results suggest that EMT and CSC properties are simultaneously regulated in the same direction. Therefore, we expected to find a positive correlation between B7-H4 and EMT-related biomarkers in ovarian serous carcinoma. However, both protein and mRNA expression of B7-H4 were significantly positively correlated with that of E-cadherin. *CDH2* expression level was inversely correlated with that of *VTCN1* in the mRNA expression data from both frozen tissue specimens and the GEPIA database. Although *VTCN1* mRNA expression level obtained from the frozen tissue specimens was not associated with that of *VIM*, *VTCN1* mRNA expression level from the GEPIA online analysis tool was inversely related to that of *VIM*. These results indicate that B7-H4 is associated with mesenchymal-to-epithelial transition (MET) in ovarian serous carcinoma. Furthermore, these results suggest that B7-H4 is strongly associated with CSC-related biomarkers, although simultaneous association with MET may be contradictory. However, the study by Jeong et al. provided an understanding of the role of B7-H4 in ovarian serous carcinoma. They showed that ESRP1 inhibited cell migration by inducing MET and stimulated cell proliferation in ovarian cancer cells [37]. In addition, Chen et al. showed that the overall survival rate and progression-free survival rate of the high ESRP1 protein-expressing group was significantly lower than that of the low ESRP1 protein-expressing group [38]. The role of B7-H4 may be similar to that of ESRP1 in epithelial ovarian cancer. Further research is needed to clarify the detailed role of B7-H4 in ovarian serous carcinoma.

Survival analysis showed the clinical significance of B7-H4 in ovarian serous carcinoma. There was no difference between the B7-H4-positive and negative tumors in terms of the overall survival rate in ovarian serous carcinomas. Liang et al. performed immunohistochemistry for B7-H4 on samples from 306 patients with ovarian serous carcinoma and investigated the clinical significance of B7-H4 expression. They showed that high B7-H4 expression was associated with high-grade serous carcinoma and advanced tumor stage, but not with overall or disease-free survival rates [39]. These results are identical to those obtained in our study. However, in our study, when survival analysis was performed only on the patients with ovarian serous carcinoma with low stromal TILs, the patients with B7-H4-positive ovarian serous carcinoma showed a significantly lower overall survival rate compared to those with B7-H4-negative ovarian serous carcinoma. The tumor suppression effect caused by increased inflammation in ovarian serous carcinoma with high stromal TILs is thought to offset the tumor progression effect caused by increased cancer stemness, which is positively related to B7-H4 expression. However, additional research is needed to identify the accurate mechanism. The patients with low stromal TIL and TIL/B7-H4-positive tumors account for approximately 77.6% and 40.3% of the total patient population, respectively (data not shown). According to our survival analysis, B7-H4 may act as a prognostic factor for patient with low stromal TIL ovarian serous carcinoma or as a therapeutic target that can suppress cancer stemness but not immune evasion in patients with low stromal TIL/B7-H4-positive ovarian serous carcinoma.

In conclusion, the positive correlation between B7-H4 expression and the density of stromal TIL indicated that B7-H4 may not be associated with host immune evasion in ovarian serous carcinoma. Our study is the first to reveal that increased B7-H4 expression is associated with cancer stemness in ovarian serous carcinoma. The low overall survival rate of B7-H4-positive ovarian serous carcinoma may be because B7-H4 expression is positively correlated with cancer stemness, leading to tumor progression. Furthermore, B7-H4 expression was strongly and positively related to E-cadherin expression at both protein and mRNA levels. The expression levels of vimentin and N-cadherin were negatively related to those of B7-H4 at the mRNA level but not at the protein level. These results suggest that B7-H4 may act as a predictive factor for patients with low stromal TIL ovarian serous carcinoma or be a treatment target for patients with low stromal TIL/B7-H4-positive ovarian serous carcinoma.

## Supplementary Information

**Additional file 1: Figure S1.** The representative cases of the tumors with low stromal TILs (A) and high stromal TILs. **Figure S2.** The representative cases of immunohistochemical expression of CSC-related biomarkers in tumor cells. CD24 (A, magnification, x200), CD44s (C, magnification, x200), CD133 (E, magnification, x200), and ALDH1 (G, magnification, x200) were immunostained on membrane or cytoplasm of ovarian serous carcinoma cells. The representative negative cases for CD24, CD44s, CD133, and ALDH1 were present in Figure B (magnification, x200), D (magnification, x200), F (magnification, x200), and H (magnification, x200), respectively. **Figure S3.** The representative cases of immunohistochemical expression of EMT-related biomarkers in tumor cells. E-cadherin (A, magnification, x200), N-cadherin (C, magnification, x200), and vimentin (E, magnification, x200) were immunostained on membrane or cytoplasm of ovarian serous carcinoma cells. The representative negative cases for E-cadherin, N-cadherin, and vimentin were present in Figure B (magnification, x200), D (magnification, x200), and F (magnification, x200), respectively.

## Data Availability

The datasets used and analyzed during the current study are available from the corresponding author on responsible request.
