# Supplementary figures and images for: The stromal tumor-infiltrating lymphocytes, cancer stemness, epithelial-mesenchymal transition, and B7-H4 expression in ovarian serous carcinoma
Source: J Ovarian Res. 2023 Jan 6;16:3. doi: 10.1186/s13048-022-01076-z (PMC9825048; doi:10.1186/s13048-022-01076-z)

# Supplementary figure S1

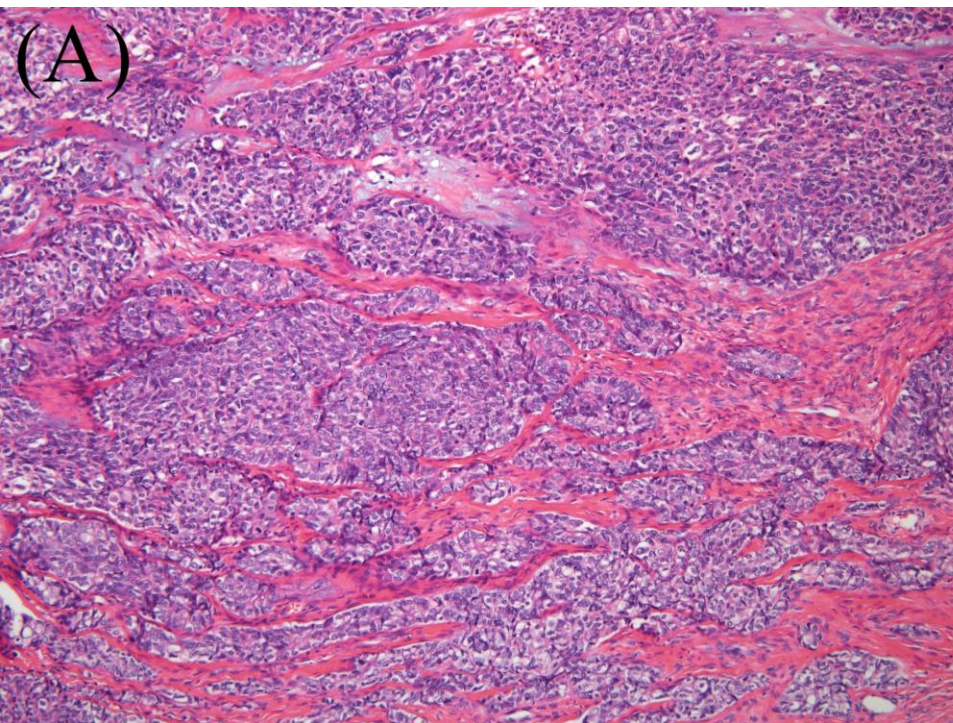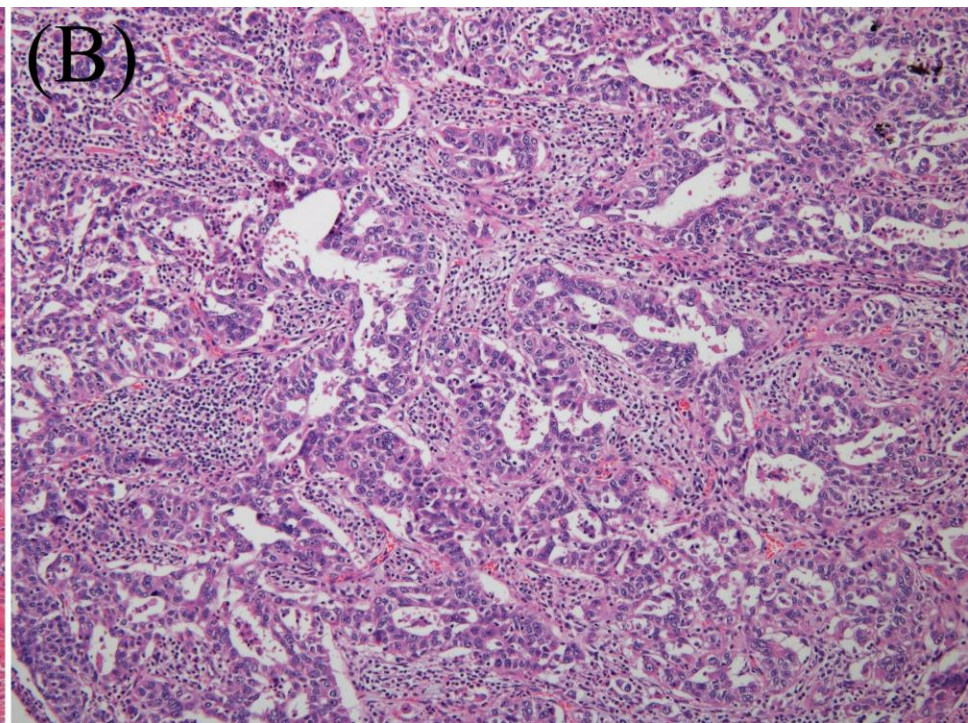

# Supplementary figure S2

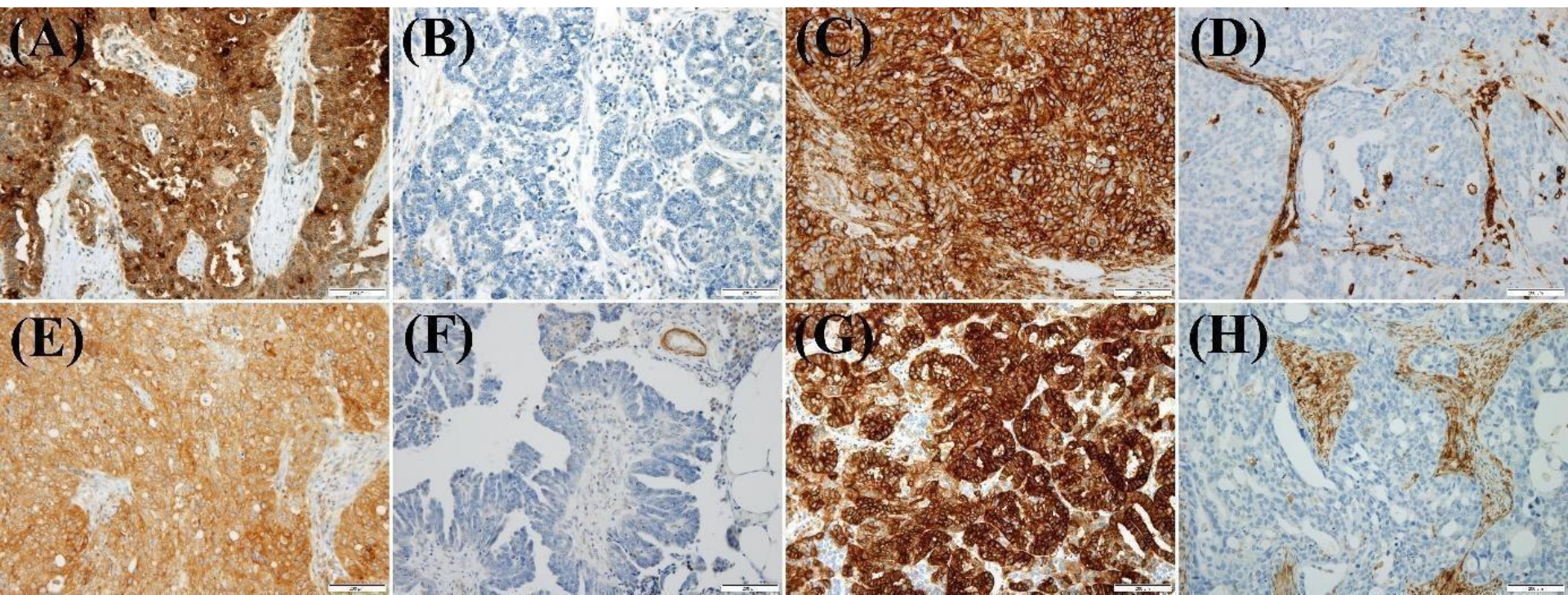

# Supplementary figure S3

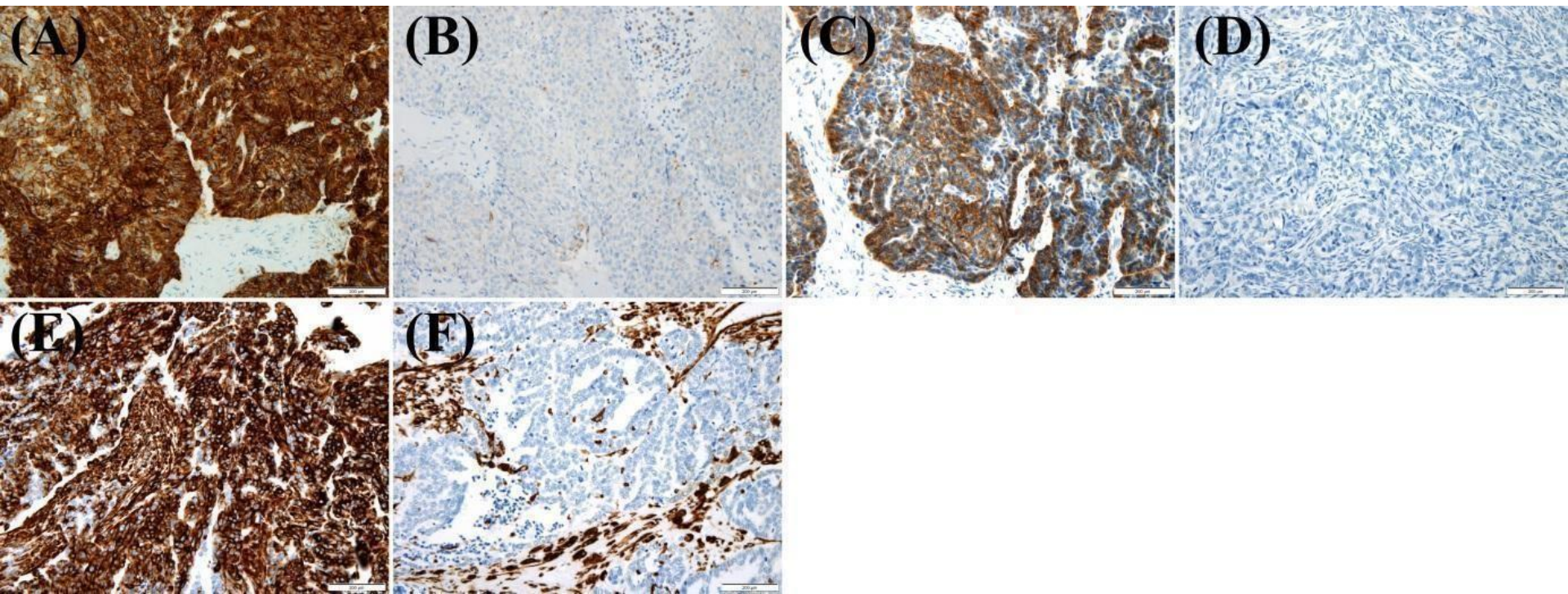

Supplement: Supplementary file 1 — Additional file 1: Figure S1. The representative cases of the tumors with low stromal TILs (A) and high stromal TILs. Figure S2. The representative cases of immunohistochemical expression of CSC-related biomarkers in tumor cells. CD24 (A, magnification, x200), CD44s (C, magnification, x200), CD133 (E, magnification, x200), and ALDH1 (G, magnification, x200) were immunostained on membrane or cytoplasm of ovarian serous carcinoma cells. The representative negative cases for CD24, CD44s, CD133, and ALDH1 were present in Figure B (magnification, x200), D (magnification, x200), F (magnification, x200), and H (magnification, x200), respectively. Figure S3. The representative cases of immunohistochemical expression of EMT-related biomarkers in tumor cells. E-cadherin (A, magnification, x200), N-cadherin (C, magnification, x200), and vimentin (E, magnification, x200) were immunostained on membrane or cytoplasm of ovarian serous carcinoma cells. The representative negative cases for E-cadherin, N-cadherin, and vimentin were present in Figure B (magnification, x200), D (magnification, x200), and F (magnification, x200), respectively. [file 13048_2022_1076_MOESM1_ESM.pdf]
